# 1,1′-[4-(4-Methoxy­phen­yl)-2,6-dimethyl-1,4-dihydro­pyridine-3,5-di­yl]diethanone

**DOI:** 10.1107/S1600536809041592

**Published:** 2009-10-17

**Authors:** M. Thenmozhi, T. Kavitha, B. Palakshi Reddy, V. Vijayakumar, M. N. Ponnuswamy

**Affiliations:** a Centre of Advanced Study in Crystallography and Biophysics, University of Madras, Guindy Campus, Chennai 600 025, India; b Organic Chemistry Division, School of Advanced Sciences, VIT University, Vellore 632 014, India.

## Abstract

In the title compound, C_18_H_21_NO_3_, which belongs to the family of calcium channel blockers, the dihydropyridine ring assumes a flattened boat conformation. The two carbonyl units adopt a synperiplanar conformation with respect to the double bonds in the dihydro­pyridine ring. The methoxy­phenyl ring is almost perpendicular to the prydine ring [dihedral angle = 89.01 (7)°]. In the crystal, the mol­ecules are connected by inter­molecular N—H⋯O hydrogen bonds.

## Related literature

For general background, see: Ganjali *et al.* (2007 ▶); Xia *et al.* (2005 ▶). For hybridization, see: Beddoes *et al.*(1986 ▶). For ring conformational analysis, see: Cremer & Pople (1975 ▶); Nardelli (1983 ▶).
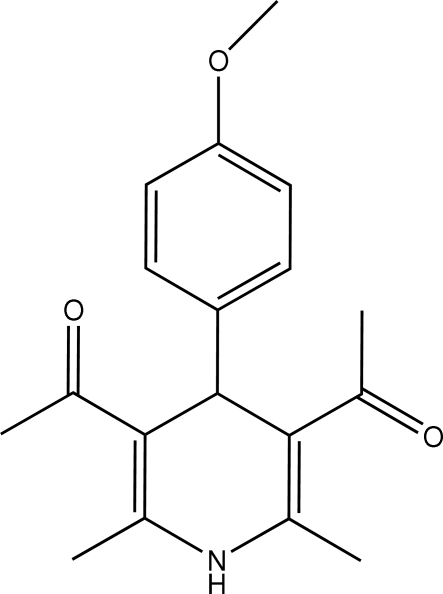


## Experimental

### 

#### Crystal data

C_18_H_21_NO_3_
*M* *_r_* = 299.36
Orthorhombic, 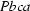

*a* = 12.0781 (3) Å
*b* = 8.9650 (2) Å
*c* = 29.3755 (8) Å
*V* = 3180.78 (14) Å^3^
*Z* = 8
Mo *K*α radiation
μ = 0.09 mm^−1^
*T* = 293 K
0.25 × 0.20 × 0.20 mm

#### Data collection

Bruker Kappa APEXII area-detector diffractometer
Absorption correction: multi-scan (*SADABS*; Sheldrick, 2001 ▶) *T*_min_ = 0.979, *T*_max_ = 0.983
36021 measured reflections
4055 independent reflections
2828 reflections with *I* > 2σ(*I*)
*R*_int_ = 0.032

#### Refinement

*R*[*F* ^2^ > 2σ(*F* ^2^)] = 0.047
*wR*(*F* ^2^) = 0.148
*S* = 1.05
4055 reflections
208 parameters
1 restraint
H atoms treated by a mixture of independent and constrained refinement
Δρ_max_ = 0.21 e Å^−3^
Δρ_min_ = −0.18 e Å^−3^

### 

Data collection: *APEX2* (Bruker, 2004 ▶); cell refinement: *SAINT* (Bruker, 2004 ▶); data reduction: *SAINT*; program(s) used to solve structure: *SIR92* (Altomare *et al.*, 1993 ▶); program(s) used to refine structure: *SHELXL97* (Sheldrick, 2008 ▶); molecular graphics: *ORTEP-3* (Farrugia, 1997 ▶); software used to prepare material for publication: *SHELXL97* and *PLATON* (Spek, 2009 ▶).

## Supplementary Material

Crystal structure: contains datablocks global, I. DOI: 10.1107/S1600536809041592/bt5060sup1.cif

Structure factors: contains datablocks I. DOI: 10.1107/S1600536809041592/bt5060Isup2.hkl

Additional supplementary materials:  crystallographic information; 3D view; checkCIF report

## Figures and Tables

**Table 1:** Hydrogen-bond geometry (Å, °)

*D*—H⋯*A*	*D*—H	H⋯*A*	*D*⋯*A*	*D*—H⋯*A*
N1—H1⋯O1^i^	0.869 (19)	2.03 (2)	2.8961 (19)	173.1 (18)

Symmetry code: (i) 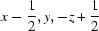.

**Fig. 1.**
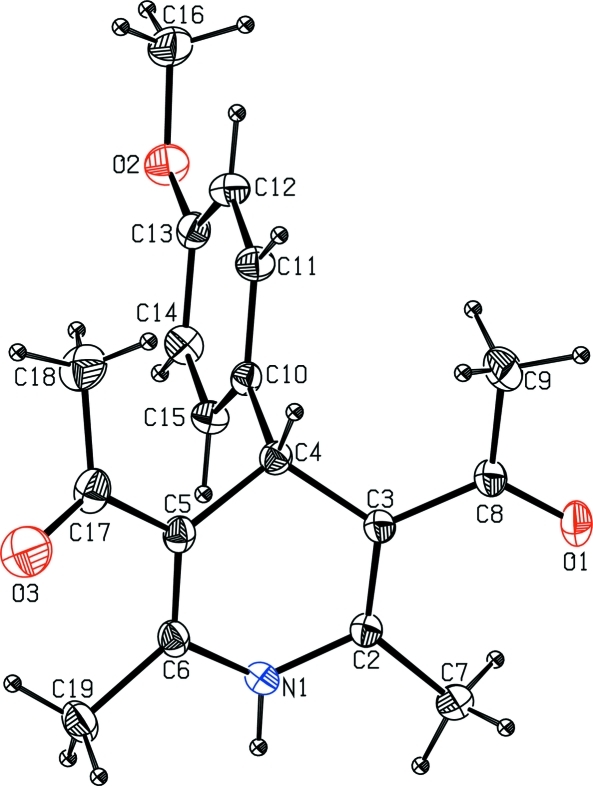
Perpective view of the molecule, showing 30% probability displacement ellipsoids.

**Fig. 2.**
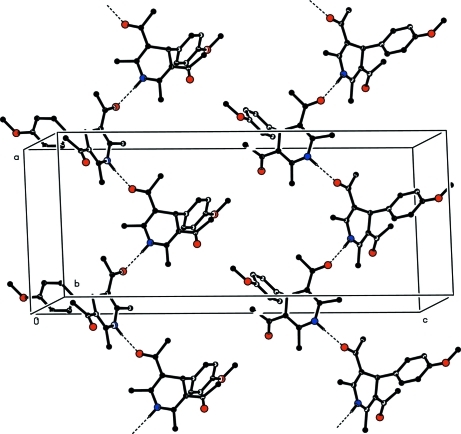
Crystal packing of the molecules viewed down a axis.

## supplementary crystallographic information

### Comment

1,4-Dihydropyridine compounds are the important class of calcium channel blockers and as such commercialized in, for instance nifedipine, amlodipine or nimodipine (Xia *et al.*, 2005). Pyridine derivatives can be used as a suitable neutral ionophore for preparing an Er(III) membrane sensor with high selectivity, which are utilized for direct monitoring of Er(III) in binary mixtures and indirect determination of fluoride ions in mouth wash preparations (Ganjali *et al.*, 2007).

The ORTEP plot of the molecule is shown in Fig. 1. The pyridine ring assumes a flattened boat conformation with puckering parameters (Cremer & Pople, 1975) q_2_= 0.2975 (16)Å, q_3_ = -0.0900 (16)Å, φ = 355.2 (3)° and asymmetry parameters (Nardelli, 1983) Δ_s_(N1,C4) = 3.26 (16)°. The methyl groups attached at C2 and C6 positions of the pyridine ring adopt equatorial oriention as can be seen from the torsion angles [C7-C2-N1-C6=]-165.71 (15)° and [C19-C6-N1-C2 =]164.36 (15)°. Both the carboxylate groups at 3^rd^ and 5^th^ positions in the pyridine ring, have synperiplanar(sp) conformation with respect to the double bonds in the dihydropyridine ring which are evident from the torsion angles [C2-C3-C8-O1=]-6.3 (3)°) and [C6-C5-C17-O3=]-17.3 (3)°. The methoxyphenyl ring is almost perpendicular to the best plane of the prydine ring as can be seen from the dihedral angle of 89.01 (7)°. The sum of the bond angles around atom N1[357.06°] of the pyridine ring is in accordance with sp^2^ hybridization (Beddoes *et al.*, 1986).

Atom N1(x,y,z) of the pyridine ring donates a proton to atom O1(-1/2+x,y,1/2-z), leading to a zig-zag chain running along the *a* - axis (Fig. 2).

### Experimental

Dimethyl-4-(4-methoxyphenyl)-2,6-dimethyl-1,4-dihydropyridine-3,5-dicarboxylate was prepared by heating the mixture of 4-methoxybenzaldehyde (10 mmol), methylacetoacetate (20 mmol) and ammonium acetate (10 mmol) at 80^o^C for 2 hours and 45 min (monitored by TLC). After completion of the reaction, the mixture was cooled to room temperature and kept for 3 days to get the solid product. The obtained solid was washed with diethyl ether and collected separately. The purity of the crude product was checked through TLC and recrystallized using acetone and ether.

### Refinement

H atoms were positioned geometrically (C-H = 0.93-0.98Å) and allowed to ride on their parent atoms, with Uiso(H) = 1.5Ueq(C) for methyl H and 1.2Ueq(C) for other H atoms.The components of the anisotropic displacement parameters of C5 and C6 in the direction of the bond between them were restrained to be equal within an effective standard deviation of 0.001.

### Crystal data

**Table:**

C_18_H_21_NO_3_	*F*(000) = 1280
*M**_r_* = 299.36	*D*_x_ = 1.250 Mg m^−^^3^
Orthorhombic, *P**b**c**a*	Mo *K*α radiation, λ = 0.71073 Å
Hall symbol: -P 2ac 2ab	Cell parameters from 4055 reflections
*a* = 12.0781 (3) Å	θ = 1.4–28.6°
*b* = 8.9650 (2) Å	µ = 0.09 mm^−^^1^
*c* = 29.3755 (8) Å	*T* = 293 K
*V* = 3180.78 (14) Å^3^	Block, light yellow
*Z* = 8	0.25 × 0.20 × 0.20 mm

### Data collection

**Table:**

Bruker Kappa APEXII area-detector diffractometer	4055 independent reflections
Radiation source: fine-focus sealed tube	2828 reflections with *I* > 2σ(*I*)
graphite	*R*_int_ = 0.032
ω and φ scans	θ_max_ = 28.6°, θ_min_ = 1.4°
Absorption correction: multi-scan (*SADABS*; Sheldrick, 2001)	*h* = −16→14
*T*_min_ = 0.979, *T*_max_ = 0.983	*k* = −12→10
36021 measured reflections	*l* = −37→39

### Refinement

**Table:**

Refinement on *F*^2^	Primary atom site location: structure-invariant direct methods
Least-squares matrix: full	Secondary atom site location: difference Fourier map
*R*[*F*^2^ > 2σ(*F*^2^)] = 0.047	Hydrogen site location: inferred from neighbouring sites
*wR*(*F*^2^) = 0.148	H atoms treated by a mixture of independent and constrained refinement
*S* = 1.05	*w* = 1/[σ^2^(*F*_o_^2^) + (0.0637*P*)^2^ + 1.0129*P*] where *P* = (*F*_o_^2^ + 2*F*_c_^2^)/3
4055 reflections	(Δ/σ)_max_ = 0.003
208 parameters	Δρ_max_ = 0.21 e Å^−^^3^
1 restraint	Δρ_min_ = −0.18 e Å^−^^3^

### Special details

**Table:**

Geometry. All esds (except the esd in the dihedral angle between two l.s. planes) are estimated using the full covariance matrix. The cell esds are taken into account individually in the estimation of esds in distances, angles and torsion angles; correlations between esds in cell parameters are only used when they are defined by crystal symmetry. An approximate (isotropic) treatment of cell esds is used for estimating esds involving l.s. planes.
Refinement. Refinement of F^2^ against ALL reflections. The weighted R-factor wR and goodness of fit S are based on F^2^, conventional R-factors R are based on F, with F set to zero for negative F^2^. The threshold expression of F^2^ > 2sigma(F^2^) is used only for calculating R-factors(gt) etc. and is not relevant to the choice of reflections for refinement. R-factors based on F^2^ are statistically about twice as large as those based on F, and R- factors based on ALL data will be even larger.

### Fractional atomic coordinates and isotropic or equivalent isotropic displacement parameters (Å^2^)

**Table:**

	*x*	*y*	*z*	*U*_iso_*/*U*_eq_	
C2	0.52089 (13)	0.12861 (18)	0.28911 (5)	0.0418 (4)	
C3	0.60545 (12)	0.18029 (17)	0.31531 (5)	0.0392 (3)	
C4	0.57919 (12)	0.24866 (17)	0.36162 (5)	0.0378 (3)	
H4	0.6343	0.3265	0.3675	0.045*	
C5	0.46584 (13)	0.32299 (17)	0.36102 (5)	0.0409 (3)	
C6	0.38706 (12)	0.27123 (18)	0.33254 (5)	0.0422 (3)	
C7	0.52978 (15)	0.0360 (2)	0.24679 (6)	0.0595 (5)	
H7A	0.5613	0.0949	0.2228	0.089*	
H7B	0.4574	0.0025	0.2379	0.089*	
H7C	0.5763	−0.0487	0.2526	0.089*	
C8	0.72005 (13)	0.1649 (2)	0.30089 (6)	0.0487 (4)	
C9	0.81050 (15)	0.2381 (3)	0.32811 (8)	0.0741 (6)	
H9A	0.8186	0.1875	0.3567	0.111*	
H9B	0.7918	0.3407	0.3334	0.111*	
H9C	0.8789	0.2327	0.3115	0.111*	
C10	0.59067 (12)	0.13184 (16)	0.39902 (5)	0.0368 (3)	
C11	0.67301 (14)	0.14207 (18)	0.43151 (5)	0.0460 (4)	
H11	0.7216	0.2225	0.4304	0.055*	
C12	0.68580 (14)	0.03681 (19)	0.46561 (5)	0.0486 (4)	
H12	0.7423	0.0467	0.4869	0.058*	
C13	0.61449 (13)	−0.08222 (17)	0.46774 (5)	0.0438 (4)	
C14	0.53161 (14)	−0.09579 (19)	0.43563 (6)	0.0478 (4)	
H14	0.4833	−0.1765	0.4368	0.057*	
C15	0.52023 (13)	0.00946 (17)	0.40189 (5)	0.0434 (4)	
H15	0.4641	−0.0015	0.3805	0.052*	
C16	0.70171 (18)	−0.1747 (2)	0.53459 (6)	0.0664 (5)	
H16A	0.7741	−0.1742	0.5211	0.100*	
H16B	0.6964	−0.2558	0.5558	0.100*	
H16C	0.6895	−0.0822	0.5502	0.100*	
C17	0.44451 (17)	0.44728 (19)	0.39277 (6)	0.0547 (5)	
C18	0.5199 (2)	0.4709 (2)	0.43225 (7)	0.0728 (6)	
H18A	0.4905	0.5480	0.4514	0.109*	
H18B	0.5918	0.5000	0.4214	0.109*	
H18C	0.5261	0.3800	0.4493	0.109*	
C19	0.26738 (14)	0.3160 (2)	0.33045 (7)	0.0589 (5)	
H19A	0.2469	0.3640	0.3584	0.088*	
H19B	0.2224	0.2289	0.3261	0.088*	
H19C	0.2564	0.3837	0.3055	0.088*	
N1	0.41402 (11)	0.16250 (16)	0.30102 (5)	0.0441 (3)	
O1	0.74715 (11)	0.09406 (19)	0.26662 (4)	0.0754 (5)	
O2	0.62054 (12)	−0.19260 (14)	0.49995 (4)	0.0596 (4)	
O3	0.36634 (16)	0.5314 (2)	0.38831 (6)	0.1011 (6)	
H1	0.3631 (16)	0.135 (2)	0.2819 (6)	0.057 (5)*	

### Atomic displacement parameters (Å^2^)

**Table:**

	*U*^11^	*U*^22^	*U*^33^	*U*^12^	*U*^13^	*U*^23^
C2	0.0386 (8)	0.0492 (9)	0.0374 (7)	0.0028 (7)	0.0024 (6)	0.0020 (6)
C3	0.0345 (7)	0.0462 (8)	0.0370 (7)	0.0005 (6)	0.0022 (6)	0.0023 (6)
C4	0.0386 (7)	0.0384 (8)	0.0364 (7)	−0.0017 (6)	0.0021 (6)	0.0010 (6)
C5	0.0446 (8)	0.0388 (8)	0.0393 (7)	0.0045 (6)	0.0071 (6)	0.0067 (6)
C6	0.0384 (7)	0.0456 (9)	0.0426 (8)	0.0069 (6)	0.0071 (6)	0.0113 (6)
C7	0.0497 (10)	0.0809 (13)	0.0479 (9)	0.0060 (9)	−0.0051 (8)	−0.0174 (9)
C8	0.0378 (8)	0.0648 (11)	0.0435 (8)	−0.0005 (7)	0.0069 (7)	0.0053 (8)
C9	0.0388 (9)	0.1061 (17)	0.0773 (13)	−0.0135 (11)	0.0059 (9)	−0.0123 (13)
C10	0.0387 (8)	0.0364 (7)	0.0351 (7)	0.0025 (6)	0.0030 (6)	−0.0027 (6)
C11	0.0473 (9)	0.0436 (8)	0.0470 (9)	−0.0054 (7)	−0.0054 (7)	0.0002 (7)
C12	0.0517 (9)	0.0502 (9)	0.0440 (8)	0.0015 (8)	−0.0097 (7)	0.0014 (7)
C13	0.0490 (9)	0.0414 (8)	0.0410 (8)	0.0093 (7)	0.0043 (7)	0.0038 (7)
C14	0.0475 (9)	0.0407 (8)	0.0552 (9)	−0.0036 (7)	0.0007 (7)	0.0042 (7)
C15	0.0416 (8)	0.0432 (8)	0.0454 (8)	−0.0025 (7)	−0.0051 (6)	0.0006 (7)
C16	0.0765 (13)	0.0739 (13)	0.0488 (10)	0.0138 (11)	−0.0043 (9)	0.0162 (9)
C17	0.0683 (12)	0.0430 (9)	0.0529 (10)	0.0123 (8)	0.0117 (8)	0.0041 (7)
C18	0.0929 (16)	0.0552 (11)	0.0704 (13)	0.0078 (11)	−0.0053 (11)	−0.0226 (10)
C19	0.0413 (9)	0.0695 (12)	0.0660 (11)	0.0145 (8)	0.0062 (8)	0.0108 (9)
N1	0.0335 (7)	0.0551 (8)	0.0438 (7)	0.0018 (6)	−0.0028 (6)	−0.0005 (6)
O1	0.0454 (7)	0.1195 (12)	0.0613 (8)	−0.0014 (8)	0.0170 (6)	−0.0231 (8)
O2	0.0696 (8)	0.0534 (7)	0.0559 (7)	0.0045 (6)	−0.0023 (6)	0.0167 (6)
O3	0.1203 (14)	0.0915 (12)	0.0916 (12)	0.0615 (11)	−0.0129 (10)	−0.0239 (10)

### Geometric parameters (Å, °)

**Table:**

C2—C3	1.360 (2)	C11—C12	1.385 (2)
C2—N1	1.3715 (19)	C11—H11	0.9300
C2—C7	1.499 (2)	C12—C13	1.373 (2)
C3—C8	1.454 (2)	C12—H12	0.9300
C3—C4	1.526 (2)	C13—O2	1.3711 (19)
C4—C5	1.523 (2)	C13—C14	1.381 (2)
C4—C10	1.524 (2)	C14—C15	1.375 (2)
C4—H4	0.9800	C14—H14	0.9300
C5—C6	1.349 (2)	C15—H15	0.9300
C5—C17	1.476 (2)	C16—O2	1.422 (2)
C6—N1	1.383 (2)	C16—H16A	0.9600
C6—C19	1.502 (2)	C16—H16B	0.9600
C7—H7A	0.9600	C16—H16C	0.9600
C7—H7B	0.9600	C17—O3	1.216 (2)
C7—H7C	0.9600	C17—C18	1.490 (3)
C8—O1	1.234 (2)	C18—H18A	0.9600
C8—C9	1.504 (3)	C18—H18B	0.9600
C9—H9A	0.9600	C18—H18C	0.9600
C9—H9B	0.9600	C19—H19A	0.9600
C9—H9C	0.9600	C19—H19B	0.9600
C10—C11	1.381 (2)	C19—H19C	0.9600
C10—C15	1.391 (2)	N1—H1	0.869 (19)
			
C3—C2—N1	119.13 (14)	C12—C11—H11	118.8
C3—C2—C7	127.17 (14)	C13—C12—C11	119.51 (15)
N1—C2—C7	113.69 (14)	C13—C12—H12	120.2
C2—C3—C8	121.17 (14)	C11—C12—H12	120.2
C2—C3—C4	119.04 (13)	O2—C13—C12	123.99 (15)
C8—C3—C4	119.72 (13)	O2—C13—C14	116.52 (15)
C5—C4—C10	113.01 (12)	C12—C13—C14	119.49 (15)
C5—C4—C3	110.63 (12)	C15—C14—C13	120.29 (15)
C10—C4—C3	110.35 (12)	C15—C14—H14	119.9
C5—C4—H4	107.5	C13—C14—H14	119.9
C10—C4—H4	107.5	C14—C15—C10	121.58 (14)
C3—C4—H4	107.5	C14—C15—H15	119.2
C6—C5—C17	121.90 (15)	C10—C15—H15	119.2
C6—C5—C4	119.39 (14)	O2—C16—H16A	109.5
C17—C5—C4	118.68 (15)	O2—C16—H16B	109.5
C5—C6—N1	119.43 (14)	H16A—C16—H16B	109.5
C5—C6—C19	127.76 (16)	O2—C16—H16C	109.5
N1—C6—C19	112.80 (15)	H16A—C16—H16C	109.5
C2—C7—H7A	109.5	H16B—C16—H16C	109.5
C2—C7—H7B	109.5	O3—C17—C5	122.42 (18)
H7A—C7—H7B	109.5	O3—C17—C18	118.06 (17)
C2—C7—H7C	109.5	C5—C17—C18	119.51 (16)
H7A—C7—H7C	109.5	C17—C18—H18A	109.5
H7B—C7—H7C	109.5	C17—C18—H18B	109.5
O1—C8—C3	122.60 (16)	H18A—C18—H18B	109.5
O1—C8—C9	117.72 (15)	C17—C18—H18C	109.5
C3—C8—C9	119.67 (15)	H18A—C18—H18C	109.5
C8—C9—H9A	109.5	H18B—C18—H18C	109.5
C8—C9—H9B	109.5	C6—C19—H19A	109.5
H9A—C9—H9B	109.5	C6—C19—H19B	109.5
C8—C9—H9C	109.5	H19A—C19—H19B	109.5
H9A—C9—H9C	109.5	C6—C19—H19C	109.5
H9B—C9—H9C	109.5	H19A—C19—H19C	109.5
C11—C10—C15	116.79 (14)	H19B—C19—H19C	109.5
C11—C10—C4	121.19 (13)	C2—N1—C6	123.26 (14)
C15—C10—C4	122.01 (13)	C2—N1—H1	116.0 (13)
C10—C11—C12	122.34 (15)	C6—N1—H1	117.8 (13)
C10—C11—H11	118.8	C13—O2—C16	116.70 (14)
			
N1—C2—C3—C8	−171.57 (15)	C5—C4—C10—C15	58.19 (18)
C7—C2—C3—C8	8.0 (3)	C3—C4—C10—C15	−66.27 (18)
N1—C2—C3—C4	11.4 (2)	C15—C10—C11—C12	−0.2 (2)
C7—C2—C3—C4	−169.03 (16)	C4—C10—C11—C12	−179.46 (15)
C2—C3—C4—C5	−30.7 (2)	C10—C11—C12—C13	−0.3 (3)
C8—C3—C4—C5	152.27 (14)	C11—C12—C13—O2	179.63 (15)
C2—C3—C4—C10	95.13 (17)	C11—C12—C13—C14	0.5 (2)
C8—C3—C4—C10	−81.92 (18)	O2—C13—C14—C15	−179.49 (15)
C10—C4—C5—C6	−95.93 (16)	C12—C13—C14—C15	−0.3 (2)
C3—C4—C5—C6	28.38 (19)	C13—C14—C15—C10	−0.2 (2)
C10—C4—C5—C17	82.28 (17)	C11—C10—C15—C14	0.4 (2)
C3—C4—C5—C17	−153.41 (14)	C4—C10—C15—C14	179.66 (14)
C17—C5—C6—N1	174.94 (14)	C6—C5—C17—O3	−17.3 (3)
C4—C5—C6—N1	−6.9 (2)	C4—C5—C17—O3	164.55 (19)
C17—C5—C6—C19	−5.9 (3)	C6—C5—C17—C18	161.94 (18)
C4—C5—C6—C19	172.25 (15)	C4—C5—C17—C18	−16.2 (2)
C2—C3—C8—O1	−6.3 (3)	C3—C2—N1—C6	13.9 (2)
C4—C3—C8—O1	170.70 (16)	C7—C2—N1—C6	−165.71 (15)
C2—C3—C8—C9	173.28 (18)	C5—C6—N1—C2	−16.4 (2)
C4—C3—C8—C9	−9.7 (2)	C19—C6—N1—C2	164.36 (15)
C5—C4—C10—C11	−122.56 (16)	C12—C13—O2—C16	4.3 (2)
C3—C4—C10—C11	112.98 (16)	C14—C13—O2—C16	−176.55 (16)

### Hydrogen-bond geometry (Å, °)

**Table:**

*D*—H···*A*	*D*—H	H···*A*	*D*···*A*	*D*—H···*A*
N1—H1···O1^i^	0.869 (19)	2.03 (2)	2.8961 (19)	173.1 (18)

Symmetry codes: (i) *x*−1/2, *y*, −*z*+1/2.

## References

[bb1] Altomare, A., Cascarano, G., Giacovazzo, C. & Guagliardi, A. (1993). *J. Appl. Cryst.* **26**, 343–350.

[bb2] Beddoes, R. L., Dalton, L., Joule, T. A., Mills, O. S., Street, J. D. & Watt, C. I. F. (1986). *J. Chem. Soc. Perkin Trans. 2*, pp. 787–797.

[bb3] Bruker (2004). *APEX2* and *SAINT* Bruker AXS Inc., Madison, Wisconsin, USA.

[bb4] Cremer, D. & Pople, J. A. (1975). *J. Am. Chem. Soc.* **97**, 1354–1358.

[bb5] Farrugia, L. J. (1997). *J. Appl. Cryst.* **30**, 565.

[bb6] Ganjali, M. R., Rezapour, M., Rasoolipour, S., Norouzi, P. & Adib, M. (2007). *J. Braz. Chem. Soc* **18**, 352–358.

[bb7] Nardelli, M. (1983). *Acta Cryst.* C**39**, 1141–1142.

[bb8] Sheldrick, G. M. (2001). *SADABS* University of Göttingen, Germany.

[bb9] Sheldrick, G. M. (2008). *Acta Cryst.* A**64**, 112–122. 10.1107/S0108767307043930 18156677

[bb10] Spek, A. L. (2009). *Acta Cryst.* D**65**, 148–155. 10.1107/S090744490804362X PMC2631630 19171970

[bb11] Xia, J. J. & Wang, G. W. (2005). *One-Pot Synthesis and Aromatization of 1,4-Dihydropyridines in Refluxing Water* in *Thieme eJournals*, Synthesis 2005, pp. 2379–2383. New York: Georg Thieme Verlag Stuttgart.

